# Benchmarking HIV Quality Measures Across US Payer Types

**DOI:** 10.1089/pop.2019.0052

**Published:** 2020-03-11

**Authors:** Julie L. Priest, Debra E. Irwin, Kristin A. Evans, Alan K. Oglesby, Brenna L. Brady

**Affiliations:** ^1^ ViiV Healthcare, Research Triangle Park, NC.; ^2^ IBM Watson Health™, Bethesda, MD.

**Keywords:** adherence, ART, health care engagement, HIV, quality measures, real world

## Abstract

Despite advances in antiretroviral therapy (ART), human immunodeficiency virus (HIV) remains a significant issue in the United States. Early diagnosis, continuous treatment access/adherence, and long-term care engagement help patients benefit fully from ART; however, a shortfall in care engagement remains, potentially leading to poorer health outcomes. This analysis benchmarks rates of health care quality and process measures to identify areas for improvement. This retrospective, claims-based, real-world cohort study assessed the percentage of prevalent (existing) and incident (newly diagnosed) patients with HIV with commercial or public health insurance meeting 4 National Quality Forum (NQF)-endorsed, 1 Pharmacy Quality Alliance (PQA), and 3 Centers for Disease Control and Prevention (CDC) measures over a 4-year period. Most prevalent patients consistently met the NQF-endorsed prescribed ART and gaps in visits measures. Longer-term visit frequency measure rates were well below the 90% Joint United Nations Programme on HIV/AIDS target. Proportion of prevalent patients meeting each NQF-endorsed measure was maintained/increased with increasing age in 2015–2016. Substantially fewer incident patients than prevalent patients met NQF-endorsed measures across all measurement periods, particularly for visit frequency (32%–51%). PQA ART adherence was low (36%–73%). CDC receipt of care rates were high (83%–92%), whereas retention in care rates were low (67%–72%) among prevalent patients. For incident patients, linkage to care rates were consistently low (21%–44%). This study benchmarks current US HIV care engagement and highlights the need for improvement in early care engagement, ART adherence and long-term retention of care among patients with HIV.

## Introduction

Human immunodeficiency virus (HIV) is a chronic acquired infection that results in the progressive loss of critical CD4 T-cells and impaired cellular immunity. If left untreated, HIV leads to the development of acquired immunodeficiency syndrome (AIDS).^1^ Prompt diagnosis and early initiation of treatment are critical to successful disease management, which focuses on both reducing viral load to undetectable levels (eg, <20–75 HIV RNA copies/mL) and enabling recovery of CD4 T-cell counts to prevent immunodeficiency.^2,3^ Periodic viral load testing is an essential part of disease management follow-up, particularly as more asymptomatic patients are treated with antiretroviral therapy (ART),^4^ as it can help assess treatment effectiveness, ensure treatment optimization, prevent the emergence of HIV drug resistance and prevent transmission to others.^4,5^

Over the last 2 decades, advances in ART have reduced rates of HIV-associated morbidity and mortality dramatically, transforming HIV from a fatal illness into a largely manageable chronic disease.^6,7^ Nevertheless, HIV remains a serious health problem in the United States from the perspectives of both transmission and management.^8^ More than 1.1 million Americans currently live with HIV, with approximately 39,000 new diagnoses annually and the highest rates being in the Southern states.^8,9^ In 2014 more than 50% of deaths (6721 of 12,333 total deaths) within the population of patients with HIV who were ever classified as having AIDS were directly attributable to HIV.^8,9^

In order for patients to benefit fully from ART and achieve optimal health outcomes, they require an early HIV diagnosis, continuous access and adherence to treatment, regular monitoring, and long-term retention of care.^10^ However, there remains a shortfall in engagement with care among patients with HIV, which may lead to poorer health outcomes.^10,11^ Studies have shown that approximately 90% of new HIV infections in the United States are contracted from patients who are either undiagnosed (∼30%) or who are diagnosed but are not engaged and/or retained in HIV care (∼60%).^12,13^ According to the Centers for Disease Control and Prevention (CDC) 2015 Prevalence-based HIV Care Continuum, 86% of all people living with HIV had an HIV diagnosis, 63% were in receipt of care (≥1 CD4 or viral load test), 49% were retained in care (≥2 CD4 or viral load tests, ≥3 months apart) and 51% achieved viral suppression (<200 copies/mL on the most recent viral load test).^14^ When applying the CDC HIV Care Continuum to people living with *diagnosed* HIV, 73% were in receipt of care, 57% were retained in care, and 60% achieved viral suppression.^14^

The Joint United Nations Programme on HIV/AIDS (UNAIDS) has proposed a 90-90-90 treatment target, whereby 90% of people with HIV will be diagnosed, on ART, and virally suppressed by 2020.^5^ Measures of care quality in HIV have been suggested by various organizations, with some overlap and some unique measures across groups. The Health Resources and Services Administration (HRSA) HIV/AIDS Bureau establishes and monitors key performance measures of care engagement and outcomes within HIV populations to ensure high-quality care, advancement along the HIV Care Continuum and to provide vital insights into HIV care and treatment, to inform future care delivery.^15^ HRSA has developed several HIV quality care measures, which have been endorsed by the US National Quality Forum (NQF).^15,16^ Similarly, the Pharmacy Quality Alliance (PQA)^17^ and the CDC^14^ also have developed measures to report on surveillance, care, and treatment for patients living with HIV.

This study aimed to utilize administrative claims data from a real-world sample of patients with HIV, in order to benchmark select quality measures and to identify key areas in need of improvement in HIV diagnosis and disease management. Although administrative claims data lack clinical data such as viral load test results typically used for surveillance, providing claims-based benchmarks may be particularly useful for tracking HIV care when these clinical data are unavailable. Electronic medical records (EMRs) may miss data in situations where patients seek care outside of the EMR system, whereas with claims data, periods of continuous enrollment can be applied to ensure that all health care services utilized during a period of time are captured. Further, the widespread availability of administrative claims databases, which are generated as billing records, also may help facilitate increased surveillance of HIV care engagement practices in the United States and be a feasible approach for US payers to monitor and improve care.

## Methods

### Study design

**FIG. 1.**
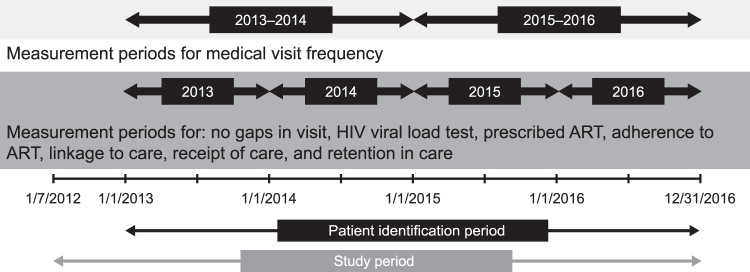
Study design. ART, antiretroviral therapy; HIV, human immunodeficiency virus.

This was a retrospective, claims-based cohort study using inpatient and outpatient data from the MarketScan Commercial Claims and Encounters, Medicare Supplemental, and Multi-State Medicaid databases, which provide access to medical and prescription drug claims for both privately and publicly insured individuals. Data spanning July 1, 2012, to December 31, 2016 were utilized, with 6 different measurement time periods defined within the full study period (Figure 1). Data are presented by measurement periods, which were defined by calendar year (from January 1) from 2013 to 2016. Data were used to assess health care engagement based on several specific NQF-, PQA-, or CDC-endorsed outcome measures.

All database records are de-identified and fully compliant with US patient confidentiality requirements, including the Health Insurance Portability and Accountability Act (HIPAA) of 1996. The databases have been evaluated and certified by an independent third party in line with HIPAA statistical de-identification standard. The databases were certified to satisfy the conditions set forth in Sections 164.514 (a)-(b)1ii of the HIPAA privacy rule regarding the determination and documentation of statistically de-identified data. Because this study used only de-identified patient records and did not involve the collection, use, or transmittal of individually identifiable data, Institutional Review Board approval to conduct this study was not necessary.

### Patient population and cohorts

Eligible patients had ≥1 non-diagnostic outpatient medical claim with an HIV diagnosis (*International Classification of Diseases, Ninth Revision, Clinical Modification* [ICD-9-CM] 042 or V08; ICD-10 B20 or Z21), or ≥1 inpatient claim with HIV as the primary diagnosis between January 1, 2013, and December 31, 2016. To allow for the evaluation of HIV experience from a payer perspective, during each measurement period patients were required to be continuously enrolled for the full calendar year and to have ≥6 months of eligibility prior to January 1 of that year. Additional eligibility criteria were required for each outcome measure (Table 1). Because of these differential eligibility criteria, not all patients qualified for each measure; therefore, resulting sample sizes reflect the maximum number of patients who could have qualified for each measure within each measurement period.

**Table 1.** Health Care Quality and Adherence Measures

Source	Measure name	Definition	Patient eligibility criteria	Measurement periods
**NQF^16^**	HIV Medical Visit Frequency	≥1 non-diagnostic, non-ER outpatient medical visit for HIV in each 6-month period over a 24-month measurement period, with ≥60 days between visits.	≥1 non-ER outpatient medical visit for HIV in the first 6 months of the measurement period, with continuous enrollment in the database for the entire measurement period and the 6 months prior.	Assessed between January 1, 2013, and December 31, 2014, and January 1, 2015, and December 31, 2016.
	Gap in HIV Medical Visits	No absence of a non-diagnostic, non-ER outpatient medical visit for HIV in the second 6 months of a given 12-month measurement period.	≥1 non-ER outpatient medical visit for HIV in the first 6 months of the measurement period, with continuous enrollment in the database for the entire measurement period and the 6 months prior.	Assessed during each full calendar year of the study (2013, 2014, 2015, 2016).
	HIV Viral Load Testing^a^	HIV viral load test ordered during the 12-month measurement period.	≥1 non-ER outpatient medical visit for HIV in the first 6 months of the measurement period, with continuous enrollment in the database for the entire measurement period and the 6 months prior.	Assessed during each full calendar year of the study (2013, 2014, 2015, 2016).
	Prescription of HIV ART	Prescription for at least 1 ART during a given 12-month measurement period.	≥1 non-ER outpatient medical visit for HIV in the first 6 months of the measurement period, with continuous enrollment in the database for the entire measurement period and the 6 months prior.	Assessed during each full calendar year of the study (2013, 2014, 2015, 2016).
**PQA^17^**	Adherence to HIV ART	Patients with ≥90% of days covered by an ART regimen in the 12-month measurement period; a variation of this outcome was ≥80% of days covered by an ART regimen.	HIV-1 diagnosis, ≥2 fills of ≥2 distinct ART agents; combination ART drugs were counted as 2 drugs.	Assessed from the date of the first ART regimen prescription fill in the measurement period to the end of the measurement period for each of the 4 full calendar years in the study.
**CDC^14^**	Receipt of care	Patients with existing (prevalent) HIV ≥13 years of age with ≥1 viral load or CD4 T-cell test within the 12-month measurement period.	Persons ≥13 years of age at the start of the measurement period, with a non-diagnostic claim for HIV in the 6 months prior to the measurement period, who were continuously enrolled in the database from the date of diagnosis to the end of the measurement period.	Assessed during each full calendar year of the study (2013, 2014, 2015, 2016).
	Retention in care	Patients with existing (prevalent) HIV ≥13 years of age and ≥2 viral load or CD4 T-cell test, ≥3 months apart, within the 12-month measurement period.	Persons ≥13 years of age at the start of the measurement period, with a non-diagnostic claim for HIV in the 6 months prior to the measurement period, who were continuously enrolled in the database from the date of diagnosis to the end of the measurement period.	Assessed during each full calendar year of the study (2013, 2014, 2015, 2016).
	Linkage to care	Newly diagnosed (within the first 3 months of each calendar year; incident patients) patients with HIV ≥13 years of age with ≥1 viral load or CD4 T-cell test within 1 month of the HIV diagnosis date.	Persons ≥13 years of age at the time of diagnosis, with continuous enrollment in the database for the entire measurement period and the 6 months prior.	Assessed during each full calendar year of the study (2013, 2014, 2015, 2016).

^a^ This measure was modified from the NQF HIV Viral Load Suppression measure because of the lack of laboratory test results in administrative claims data and reports the proportion of patients who had evidence of a viral load test being performed.

ART, antiretroviral therapy; CDC, Centers for Disease Control and Prevention; ER, emergency room; HIV, human immunodeficiency virus; NQF, National Quality Forum; PQA, Pharmacy Quality Alliance.

The study population was divided into cohorts based on insurance program (commercial/Medicare [where Medicare patients were ≥65 years of age], or Medicaid) and HIV experience at the start of each measurement period (prevalent or incident patient). Prevalent patients were defined as eligible patients with a non-diagnostic claim containing an HIV diagnosis any time before January 1 of a given year. Once a patient was designated as prevalent, he/she could not be classified as an incident patient in subsequent calendar years. Although incident patients were not an identified population in the measures selected for this study, they were included to offer a cross-sectional snapshot of how these measures could be assessed in newly diagnosed patients. Incident patients were defined as eligible patients who did not have evidence of a non-diagnostic claim containing an HIV diagnosis prior to January 1 of a given year and who had their first HIV diagnosis within the first 3 months (January 1 to March 31) of that year. Patients who had evidence of their first HIV diagnosis after March 31 of a given calendar year were not included in the analyses for that year. Once a patient was designated as incident, he/she could not be classified as an incident patient in subsequent calendar years; however, he/she could be reclassified as a prevalent patient in subsequent calendar years.

### Outcomes

Four NQF-endorsed measures, 1 PQA measure, and 3 CDC measures were assessed between 2013 and 2016 (Table 1). For the NQF-endorsed measures (HIV Medical Visit Frequency, Gap in HIV Medical Visits, HIV Viral Load Testing, and Prescription of HIV ART), the proportion of all qualifying patients who met each measure during each measurement period were described for both prevalent and incident patients for each database. For the NQF Gap in HIV Medical Visits measure, the number of prevalent and incident patients with HIV who did not have a gap in medical visits was reported. Because of the lack of clinical laboratory results data in the claims databases, the Viral Load Testing measure was modified from the NQF Viral Load Suppression Measure, which examines the percentage of patients with a viral load ≤200 copies/mL, and instead reports the proportion of patients who had evidence of a viral load test being performed. The measurement periods for the NQF-endorsed measures were each of the 4 full calendar years within the study (ie, 2013, 2014, 2015, 2016), with the exception of the HIV Medical Visit Frequency*,* which was assessed over 2 longer measurement periods, from January 1, 2013, to December 31, 2014 and from January 1, 2015, to December 31, 2016.

For the PQA Adherence to HIV ART measure, the number and proportion of patients who were classified as adherent to ART (proportion of days covered [PDC] ≥0.90) was assessed for prevalent and incident patients in each database. PDC was calculated as the total number of days the patient was covered by ≥2 distinct ARTs, based on the prescription fill dates and days of supply, divided by the number of days between the first fill date and the end of the measurement period. Each individual drug in a combination ART counted as a distinct ART. The measurement periods were each of the 4 full calendar years within the study.

During each measurement period, the proportions of qualifying patients who met the CDC Receipt of Care and Retention in Care measures were described for prevalent patients in each database, whereas those who met the CDC *Linkage to* Care measure were described for incident patients in each database. Again, the measurement periods were each of the 4 full calendar years within the study.

### Statistical analysis

Analyses were descriptive with no specific hypothesis tests conducted. Distributions of continuous variables were summarized using means and standard deviations (SD), with frequencies and percentages being reported for categorical variables. Demographic characteristics were assessed at the beginning (January 1) of each measurement period among all prevalent and incident patients with HIV, and within groups defined by meeting the NQF and CDC measures.

## Results

### Patient demographics

Baseline patient demographics for 2016 are described in Table 2 and had a similar distribution to those observed in previous study years (data not shown). In 2016, there were 23,937 prevalent patients with commercial/Medicare insurance (95.9% commercial; 4.1% Medicare) and 9261 with Medicaid insurance. There were 737 incident patients with commercial/Medicare insurance (95.3% commercial; 4.7% Medicare) and 489 with Medicaid insurance. Patients with commercial/Medicare insurance were more likely to be male than patients with Medicaid insurance for both prevalent and incident patients (Table 2). Prevalent patients were generally older than incident patients in both the commercial/Medicare (mean [SD]: 48.0 [11] vs 41.1 [15] years) and the Medicaid (mean [SD]: 44.7 [13] vs 39.0 [15] years) insurance plans. Both prevalent and incident patients with Medicaid insurance were predominantly black (67% and 66%, respectively). Patients with commercial/Medicare insurance most often resided in the Southern region of the United States (53% and 59% for prevalent and incident patients, respectively). Over the 4 years of analysis, there was a trend toward an increased percentage of incident patients with commercial/Medicare insurance being female or from the South (female: 20% in 2013 vs 24% in 2016; South: 45% in 2013 vs 59% in 2016), whereas for Medicaid insurance, there was a trend toward an increase in the percentage of incident patients being male (39% in 2013 vs 50% in 2016). These trends were not observed for prevalent patients in either database.

**Table 2.** Patient Demographics for the 2016 Measurement Period Population^a^

	Commercial/Medicare plan		Medicaid plan	
	Prevalent (n = 23,937)	Incident (n = 737)	Prevalent (n = 9261)	Incident (n = 489)
Age, mean (SD)	48.0 (11.4)	41.1 (14.6)	44.7 (12.5)	39.0 (14.7)
Age group, n (%)				
<18 years	109 (0.5)	13 (1.8)	303 (3.3)	34 (7.0)
18–34 years	3170 (13.2)	265 (36.0)	1686 (18.2)	151 (30.9)
35–44 years	4534 (18.9)	138 (18.7)	1848 (20.0)	92 (18.8)
45–54 years	9003 (37.6)	187 (25.4)	3187 (34.4)	134 (27.4)
55–64 years	6139 (25.6)	99 (13.4)	2218 (23.9)	77 (15.7)
≥65 years	982 (4.1)	35 (4.7)	19 (0.2)	1 (0.2)
Male, n (%)	19,737 (82.5)	561 (76.1)	4449 (48.0)	244 (49.9)

^a^ Assessed on January 1, 2016 unless otherwise stated.

SD, standard deviation.

### NQF-endorsed measures: prevalent patients

**FIG. 2.**
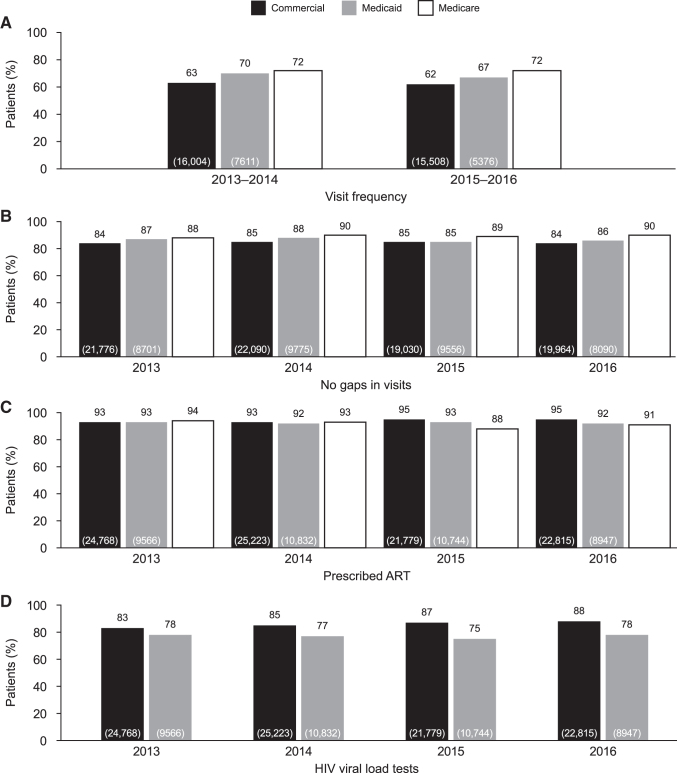
Proportion of prevalent patients with commercial, Medicare^a^ or Medicaid insurance achieving NQF-endorsed measures. ^a^Viral load test claims data were determined to be incomplete for patients with Medicare insurance plans because of billing/reimbursement practices between Medicare and Supplemental Medicare. Values in *brackets* indicate total number of patients in each group (N) who were eligible for the measure. ART, antiretroviral therapy; HIV, human immunodeficiency virus, NQF, National Quality Forum.

Overall, the proportion of prevalent patients meeting the NQF HIV Medical Visit Frequency measure was well below the 90% UNAIDS target,^5^ irrespective of insurance cohort or measurement period assessed (62%–72%; Figure 2A). For the commercial, Medicare, and Medicaid cohorts, the majority of prevalent patients assessed met the Gap in HIV Medical Visits (84%–90%) and Prescription of HIV ART (91%–95%) measures (Figure 2B and 2C). The majority of prevalent patients with commercial insurance met the HIV Viral Load Testing measure; however, rates were lower for prevalent patients with Medicaid insurance (Figure 2D). For both the HIV Medical Visit Frequency and Gap in HIV Medical Visits measures, proportions of patients meeting the measures were highest in patients with Medicare insurance, followed by those with Medicaid insurance (Figures 2A and 2B). The proportion of patients meeting the Prescription of HIV ART measure were similar in 2013 and 2014 regardless of insurance plan, although in 2015 and 2016 the proportion of patients meeting this measure was highest in patients with commercial insurance (Figure 2C). The proportion of patients who met the HIV Viral Load Testing measure was higher for those with commercial insurance than Medicaid insurance; no data were available for patients with Medicare insurance (Figure 2D). For all measures with available data, the proportion of patients with either Medicare or Medicaid insurance meeting the measures showed no clear pattern of improvement over the 4 years of analysis; similarly, there was no clear pattern over time for patients with commercial insurance (Figure 2).

**FIG. 3.**
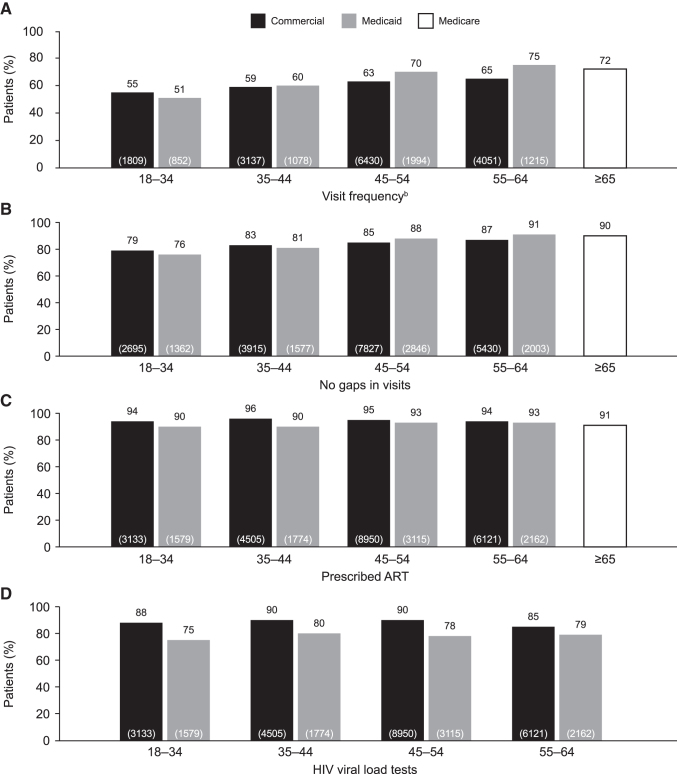
Proportion of patients achieving NQF-endorsed measures analyzed by age group (years) for prevalent patients with commercial/Medicare and Medicaid insurance^a^ in 2016. ^a^Data for patients ≥65 years of age with Medicaid insurance are not presented because of low sample size. ^b^Visit frequency was assessed over a 24-month measurement period (2015–2016). Values in *brackets* indicate total number of patients (N) in each group who were eligible for the measure. ART, antiretroviral therapy; HIV, human immunodeficiency virus, NQF, National Quality Forum.

An analysis of NQF-endorsed outcome measures by age in adult prevalent patients in 2016 revealed a trend for a maintained or an increased proportion of patients meeting each of the measures with increasing age (Figure 3). The proportion of patients meeting the HIV Medical Visit Frequency measure was lower for younger patients (18–44 years of age) compared with older patients (45–64 years of age) for both those with commercial insurance and those with Medicaid insurance (Figure 3A). Similarly, the proportion of patients with either commercial or Medicaid insurance who met the Gap in HIV Medical Visits measure also was lower for younger patients compared with older patients (Figure 3B). The proportion of patients meeting the Prescription of HIV ART and HIV Viral Load Testing measures were similar across age groups for both the commercial and Medicaid insurance cohorts (Figures 3C and 3D).

### NQF-endorsed measures: incident patients

Compared with prevalent patients, the proportions of incident patients meeting all measures were substantially lower across all measurement periods. These were lowest for HIV Medical Visit Frequency*,* with only 40%–41% of patients with commercial/Medicare insurance and 32%–51% of patients with Medicaid insurance meeting this outcome (data not shown).

### PQA PDC for ART

**FIG. 4.**
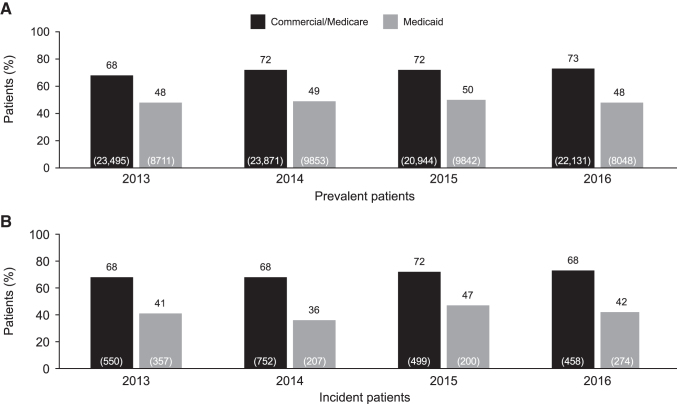
Proportion of prevalent **(A)** and incident **(B)** patients with PQA PDC for ART ≥90% by commercial/Medicare and Medicaid insurance status. Values in *brackets* indicate total number of patients in each group (N) who were eligible for the measure. ART, antiretroviral therapy; PDC, proportion of days covered; PQA, Pharmacy Quality Alliance.

The proportion of patients meeting the PQA Adherence to HIV ART (PDC for ART ≥90%) was low for patients with commercial/Medicare insurance and Medicaid insurance for both prevalent (commercial/Medicare: 68%–73%; Medicaid: 48–50%) and incident (commercial/Medicare: 68%–72%; Medicaid: 36%–47%) patients (Figure 4). Rates of adherence to ART for prevalent patients with commercial/Medicare insurance increased steadily by 5% over the study period, from 2013 to 2016.

When adherence to ART was considered as PDC ≥80%, the proportion of patients with commercial/Medicare insurance who met this target was 81%–84% for prevalent patients and 79%–82% for incident patients. For patients with Medicaid insurance, the proportions who met this measure were 62%–64% and 53%–63% for prevalent and incident patients, respectively.

### CDC measures

Despite methodological differences, the proportions of prevalent patients meeting the CDC measures were similar to those who met the NQF HIV Medical Visit Frequency measure, with 67%–68% and 67%–72% of patients with commercial/Medicare and Medicaid insurance, respectively, meeting the Retention in Care measure. The rates of prevalent patients meeting the Receipt of Care measure were high at 83%–88% and 90%–92% for patients with commercial/Medicare and Medicaid insurance, respectively, whereas the proportions of incident patients meeting the Linkage to Care measure were low, both in patients with commercial/Medicare insurance and in those with Medicaid insurance (41%–44% and 21%–38%, respectively).

## Discussion

This retrospective, real-world, US claims-based study provides a benchmark for rates of health care engagement and treatment adherence in patients with HIV, allowing key areas to be identified that may help improve disease management processes. In particular, the study shows an overall lack of engagement with care and poor adherence to ART, particularly in younger people and incident patients recently diagnosed with HIV.

Early engagement with outpatient care following an HIV diagnosis, and subsequent retention in care, has been shown to be predictive of positive clinical outcomes in patients with HIV^18,19^ and also is associated with faster and greater viral suppression and reduced viral load burden.^20,21^ In this study, rates of meeting the CDC Linkage to Care measure were low among incident patients with HIV (21%–44%), suggestive of poor engagement in care in the year following diagnosis, and putting them at risk of poor clinical outcomes. Although the NQF-endorsed measures were developed for prevalent patients, the low proportion of incident patients meeting the HIV Medical Visit Frequency and Gaps in Medical Visits measures also is noteworthy, given that poor visit adherence during the first 2 years in care is associated with greater viral loads.^20–22^ In addition, patients with a gap in their engagement in care are more likely to experience future gaps in care,^22^ with studies also showing that each missed clinic visit conveys a 17% increased risk of delayed viral suppression.^21^ Therefore, this study highlights a particular need for greater health care engagement and increased interaction with health care providers among newly diagnosed patients in the first year following their HIV diagnosis, in order to reduce the risk of poor clinical outcomes. These same principles apply to younger people living with HIV who were shown here to also have low rates of meeting the HIV Medical Visit Frequency and Gaps in Medical Visits measures. Retaining younger patients in care and maintaining viral suppression can improve long-term outcomes with reduced morbidity and mortality.^20^

In addition to early linkage and retention in care, adherence to ART is critical for both achieving and maintaining virologic suppression.^23–25^ Previous ART nonadherence and unplanned treatment interruptions of ≥48 hours also have been demonstrated to be predictors of virologic failure, (≥2 viral load tests of >400 copies/mL),^26^ and low adherence to ART may lead to poorer patient outcomes, such as an increased risk of hospitalization.^27^ In this study, rates of adherence to ART were low in both prevalent and incident patients, particularly for patients enrolled in the Medicaid database (36%–50%). These findings indicate a substantial need to improve patient compliance with ART treatment guidelines as well as targeted interventions to improve medication adherence.

The Department of Health and Human Services (DHHS) guidelines for the use of ART detail methods to improve adherence to ART for patients with HIV.^3^ The success of ART adherence programs requires a coordinated effort of a multidisciplinary health care team, including clinicians, support workers, case managers, pharmacists, and social workers, as well as a trusting relationship with the patient. These guidelines state that it is critical that patients are provided with the knowledge to fully understand all aspects of their HIV infection, including the course of disease, clinical parameters and expected outcomes, the importance of the HIV care continuum,^14^ and the potential consequences of poor adherence to ART.^3^ To achieve better adherence to therapy, the DHHS guidelines suggest involving patients in the ART regimen selection process. This is important to tailor a patient's ART regimen to their particular needs; for example, taking into consideration potential side effects, comorbidities, dosing schedule, pill burden, and food requirements.^3,28^ In addition, prior to initiating ART, it is important to assess the patient's potential barriers to adherence, and to provide the necessary resources and medication management skills to help address these barriers, such as help finding resources to assist with treatment costs and transport, or providing counseling to overcome stigma, substance use, or depression. This tailored process should continue throughout the patient's ART program, ideally assessing adherence at each clinic visit and reassessing the patient's situation and any potential barriers.^3^

In addition to improving clinical outcomes for patients with HIV, prompt diagnosis, adherence to treatment, and achieving and maintaining viral suppression are essential for reducing transmission of HIV and preventing new infections.^12,13^ In the absence of a cure, this is the only path toward ending the HIV and AIDS epidemic that, despite vast improvements over the last few decades, still represents a significant health burden in the United States today.^8,9^ To reach the UNAIDS 90-90-90 treatment target of 90% of people with HIV being diagnosed, on ART, and virally suppressed by 2020,^5^ it is essential that health care providers and payers are aware of the current situation of HIV health care engagement. This study provides a benchmark of the current status of HIV care engagement in the United States and identifies key areas in need of improvement — namely, the need for improved early engagement in care, adherence to ART, and long-term retention of care among patients living with HIV. Moreover, the measures defined in this study also would allow payers to examine quality performance with the claims data they have readily available.

This study is not without limitations, including those inherent in any retrospective analysis based on administrative health care claims. For example, administrative claims lack clinical detail such as laboratory results data and are subject to data coding limitations or data entry error. Additionally, the PDC for ART measure quantifies the proportion of days in the period of interest that a patient has access to ART medications based on filled prescriptions; it is assumed that patients took their medications as prescribed, though actual medication consumption cannot be confirmed. Similarly, this claims-based analysis assumes that diagnosis codes on medical claims accurately represent a patient's disease status, though laboratory values or other diagnostic testing results are not available to confirm the presence of actual disease. In addition to the limitations inherent to a retrospective analysis, it also should be noted that when assessing modified NQF-endorsed measures for incident patients, the sample size was too small to warrant a stratified presentation of results by age group. Consequently, these data have not been shown. Results of this analysis may not be generalizable to patients with HIV with insurance not covered by the databases utilized, who have no health insurance, or are not continuously enrolled in an insurance plan for various reasons; however, this study may provide insights that are applicable in such situations. For the results in the Medicaid population, because of confidentiality reasons, the states that contribute to this data source are unknown so this population cannot be described geographically. Finally, limitations of the Medicare billing and reimbursement processes could explain the low rate of viral load and/or CD4 T-cell testing observed among patients ≥65 years of age (data not shown), rather than actual rates of measure compliance among the Medicare population, and should be considered for future studies.

## Conclusions

This study provides a benchmark of the current state of health care engagement among people living with HIV in the United States. Despite some areas of high compliance with existing health care quality measures, this study highlights key areas in which work is still required to improve patient outcomes—namely, long-term health care engagement and adherence to ART, particularly in younger people living with HIV and newly diagnosed patients with HIV across health care insurance plans. This study provides a starting point for health care providers and payers to begin seeking effective methods to address current limitations in health care delivery to people with HIV. This will be critical to improve patient outcomes and work toward the UNAIDS 90-90-90 treatment goal.
